# Responding to warming in polar oceans: A commentary on Molina et al. (2022)

**DOI:** 10.1111/gcb.16468

**Published:** 2022-10-17

**Authors:** Lloyd Samuel Peck

**Affiliations:** ^1^ British Antarctic Survey Cambridge UK

## Abstract

Antarctic marine species live in one of the most thermally stable environments on Earth. They have evolved in these cold stable conditions for many millions of years. The long period for evolution, the isolation and mixing of populations produced by glacial cycles and the environmental heterogeneity in terms of light, productivity and physical disturbance, has produced a diverse fauna with an estimated 20 000 species, or more, living on the seabed. It has also produced a fauna that is possibly the most sensitive to warming on Earth in an environment that is changing faster than most, if not all, others. There is a great need to understand this threatened biodiversity and to find ways to mitigate the future prospects of species loss in this special environment that supports unique biology including the only vertebrate species on Earth that live without haemoglobin.

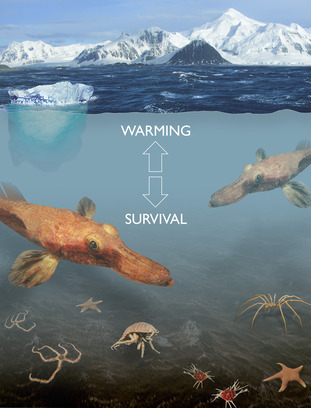

One of the most pressing questions for climate change biologists is to understand how species can and will respond to altered environments. Quantifying impacts across scales from genes through individuals to communities is key to assessing the effects of climate change on biodiversity. Understanding abilities to respond to change, the mechanisms involved, and which species are more and less resistant is crucial to achieving this aim (Palumbi et al., 2019).

Two main approaches have been used to assess the effects of change: post‐hoc observations of responses in wild populations correlated with alterations to environments; and detailed observations of organism capabilities in manipulated conditions in experiments. The former are used to predict distributions based on future environments, but misses phenotypic plasticity, life histories, ecological interactions and dependencies, and geographical barriers. The latter misses much ecology, environmental variation, and longer term, across generational plasticity because most research is within the laboratory and short timescale, although recent approaches have begun to conduct more in situ manipulations, even in marine environments (Ashton et al., 2017). There is consensus that predictive modelling incorporating both is needed for accurate forecasting (Chown & Gaston, 2016).

Phenotypic plasticity works within the generation, primarily through acclimation. It also works across generations, where both habituation of F_1_ parental broodstock during gametogenesis and developmental plasticity of embryos, larvae, and juveniles can have very large effects. In the absence of data on factors such as acclimation and developmental plasticity, data obtained are primarily limited to acute responses to altered conditions and much of the overall potential response is missed. Acclimation and trans‐generational effects probably will have the largest effect on responses to climate change, and the literature is noticeably sparse on these factors (but e.g. see Rodríguez‐Romero et al., 2016).

Temperature is recognized to have the largest effect on biological systems as a factor in climate change in the vast majority of scenarios (Clarke, 2017). The amount of warming that marine animals can survive in experiments varies across the globe. In general, species from environments with little temperature variation (the tropics and the polar regions) appear to have little capacity and are stenothermal, whereas those from more variable environments appear more resilient. This has most efficiently been measured by comparing the upper temperature limits (critical thermal maximum [CT_max_]) of animals in warming experiments. These types of experiments may be performed using different ramping rates (e.g. 1°C per hour, 1°C per day). The experienced thermal envelope of the animal under study and the difference in their CT_max_ is seen as a thermal buffer to warming and is called either the warming tolerance or warming allowance (Peck, 2018). The warming allowance for tropical and polar species is around 3–4°C, but for warm temperate, temperate and cool temperate, it is 6–10°C. Polar species have been recognized as being stenothermal since the 1960s when Antarctic fish were first demonstrated to be unable to survive at 6°C (Somero & DeVries, 1967). Research since then has confirmed that Antarctic fish are stenothermal, and that some are even less capable of resisting warming than reported by Somero and DeVries (1967). Work has also shown that species living on the slightly warmer Antarctic Peninsula are more tolerant of warming than those from high polar sites such as McMurdo Sound, and that marine invertebrates appear less tolerant of warming than fish (Peck, 2018).

A significant confounding factor in understanding thermal tolerance, temperature limits, and metrics such as the CT_max_ is that upper temperature limits in experiments vary substantially with the rate of warming in the trials. The change in limits can make interpretation difficult as in some Antarctic species warming at 1°C per hour can produce limits above 20°C, whereas warming at 1°C every 2 or 3 months produces limits around 3–4°C. There have also been two approaches to experiments aimed at evaluating thermal limits, those with continuous ramping and those that employ a rapid acute warming to a set temperature and then evaluate the time required before animals become unresponsive, termed tolerance landscapes. The two approaches produce similar, but slightly different estimates of CT_max_ and there is debate over what the differences between the methods represent (e.g. Rezende et al., 2014). Despite this it is possible to recalculate CT_max_ from one approach to the other, as the underlying physiological and molecular mechanisms setting the limits are the same (Morley et al., 2016).

The article presented in this issue by Molina et al. (2022) takes this field forwards in an interesting way by conducting a meta‐analysis of published thermal limit data for Antarctic marine species and analysing how survival is impacted by both intensity and duration of a temperature insult. Using 184 assessments of thermal limit for 39 species, they brought together data from continuous ramping and tolerance landscapes to show that Antarctic species are currently experiencing temperatures that are likely physiologically stressful and that marine communities are already impacted and vulnerable to present and future warming. In a new approach, the authors use thermal death time (TDT) curves to allow them to estimate CT_max_ and *z*, the temperature sensitivity constant, for different phylogenies from endpoint data. Unsurprisingly, Antarctic species had lower thermal tolerances than species from lower latitudes. However, the new approach allowed Molina et al. (2022) to identify differences between phylogenetic groups and to produce values for an ‘average species’ within a taxon. Arthropods and fish had the lowest *z*, with values of 1.3 and 2.1, respectively, molluscs (4.5) and echinoderms (6.1) were in the mid‐range and brachiopods had the highest *z* at 8.7. There were thus very strong differences in sensitivity between groups, and it appears this approach might be particularly efficient in identifying such differences in sensitivities.

The TDT approach also allowed the authors to compare CT_max_, the absolute thermal limit with *z*, sensitivity to warming. This allowed them to identify sensitivity to different scenarios of rapid warming, for example, as experienced in heatwaves compared to the more gradual chronic warming often visualized by researchers investigating responses to future climate change. In this respect, the authors were able to show arthropods and fish will likely be more strongly impacted by future rapid acute warming events, whereas brachiopods, echinoderms, and molluscs will be more affected by chronic warming scenarios.

Molina et al. (2022) have brought a new approach to the evaluation of thermal tolerance experiments that throws new light on how to interpret data from experiments, and has the possibility of bridging the gaps in the field and allowing better understanding from the ability to synthesize across approaches. The data are still limited and there are gaps, which means conclusions still need to be interpreted with care. However, this paper should provide impetus to other research groups, across the globe, to conduct experiments across a wider range of taxonomic groups so that the most vulnerable taxa can be identified. Then the real aim can be addressed of identifying those species most likely to disappear and whether they play key roles that are essential for future ecosystem persistence.

## Data Availability

No new data in manuscript.
